# Dual Challenges: Addressing Post-Traumatic Retroperitoneal Urinoma in the Context of Pyeloureteral Duplication

**DOI:** 10.3390/diagnostics16081132

**Published:** 2026-04-09

**Authors:** Marius Doru Stan, Irina Vlase, Emma Gheorghe, Bogdan Alexandru Georgescu, Dragos Fasie, Mihaela Botnarciuc, Lucian-Flavius Herlo, Ionut Ciprian Iorga, Felix Voinea, Andreea Nelson Twakor, Bogdan Cimpineanu, Iulian Catalin Bratu

**Affiliations:** 1 Department of Urology, County Clinical Emergency Hospital of Constanta, 900591 Constanta, Romania; marius.stan@365.univ-ovidius.ro (M.D.S.); ionut.iorga@365.univ-ovidius.ro (I.C.I.); felix.voinea@365.univ-ovidius.ro (F.V.); 2 Faculty of General Medicine, “Ovidius” University, 900470 Constanta, Romania; emma.gheorghe@365.univ-ovidius.ro (E.G.); orl.dr.alexgeorgescu@gmail.com (B.A.G.); mihaela.botnarciuc@365.univ-ovidius.ro (M.B.); dr_bratuiulian@yahoo.com (I.C.B.); 3 Department of Dermato-Venerology, County Clinical Emergency Hospital of Constanta, 900591 Constanta, Romania; 4 Department of Hemodialysis, County Clinical Emergency Hospital of Constanta, 900591 Constanta, Romania; 5 Department of Blood Transfusions, County Clinical Emergency Hospital of Constanta, 900591 Constanta, Romania; bogdan.cimpineanu@365.univ-ovidius.ro; 6 Doctoral School, “Victor Babes” University of Medicine and Pharmacy, 300041 Timisoara, Romania; flavius.herlo@umft.ro; 7 Department of Internal Medicine, County Clinical Emergency Hospital of Constanta, 900647 Constanta, Romania; an-dree.purcaru@365.univ-ovidius.ro

**Keywords:** traumatic retroperitoneal urinoma, pyeloureteral duplication, ureteral reimplantation, ureteroscopy, Uro-CT

## Abstract

Background and Clinical Significance: Retroperitoneal urinomas are uncommon complications that can arise following trauma, particularly in the context of congenital anomalies such as pyeloureteral duplication. These conditions pose significant diagnostic and therapeutic challenges, requiring a comprehensive and multidisciplinary approach to ensure optimal patient outcomes. Case Presentation: Here, we report the case of a 22-year-old male who presented to the emergency department with right lumbar and flank pain, nausea, and abrasions following a fall from a height. Initial imaging revealed a right-sided retroperitoneal urinoma and a rare congenital anomaly: complete pyeloureteral duplication with the upper pole draining into the right seminal vesicle. The patient underwent two surgical interventions, including the insertion of a ureteral stent and reimplantation of the ureter using a latero-terminal U trans U technique. Conclusions: This case highlights the complexity of managing traumatic retroperitoneal urinomas associated with congenital anomalies such as complete pyeloureteral duplication. It emphasizes the importance of timely surgical intervention to prevent complications and improve patient outcomes.

## 1. Introduction

Retroperitoneal urinomas, though rare, represent a serious complication of trauma, particularly in cases involving the urinary tract. These conditions arise when urine leaks into the retroperitoneal space, often due to injury to the renal pelvis or ureter [1]. The presence of congenital anomalies, such as pyeloureteral duplication, can further complicate the clinical picture, making diagnosis and management more challenging. Early detection through imaging and timely surgical intervention are crucial to prevent complications such as infection, fibrosis, or impaired renal function [2].

Renal trauma represents a relatively uncommon entity within the overall spectrum of traumatic injuries. It accounts for approximately 1–5% of all trauma admissions, although it is the most frequently injured organ within the genitourinary tract [3]. Most cases occur following blunt abdominal trauma and are typically associated with high-energy mechanisms such as road traffic accidents or falls [4].

In the context of renal trauma, injuries involving the collecting system may lead to urinary extravasation and subsequent urinoma formation. Reported rates of urinary leakage vary widely depending on injury severity and imaging protocols, with estimates ranging from approximately 1–7% of all renal injuries, while higher incidences are observed in high-grade injuries involving the collecting system [5].

Ureteral duplication, also known as a duplex collecting system, is the most common congenital anomaly of the urinary tract, with an estimated prevalence of approximately 0.8–1% of the general population [6]. This anomaly can occur in two forms: incomplete duplication, in which both ureters merge before entering the bladder, and complete duplication, where two independent ureters drain separately [7].

Complete duplication is more frequently associated with complications such as ureteropelvic junction obstruction, ectopic ureteral insertion, hydronephrosis, and recurrent infections [8]. Ectopic ureteral insertion occurs predominantly in females; in males it is considerably rarer and most commonly involves the prostatic urethra, ejaculatory duct, or seminal vesicle. Insertion into the seminal vesicle represents an exceptionally rare anatomical variant, with only isolated cases described in the literature [9].

Imaging studies, especially contrast-enhanced CT scans, play a key role in identifying the extent of injury and guiding treatment decisions [10]. Management strategies involve surgical intervention, which can range from minimally invasive procedures to more complex reconstructive surgeries, depending on the severity of the injury and the presence of congenital anomalies [11].

These variances are relatively rare and can be asymptomatic until an injury or other condition brings them to light. The presence of such anomalies necessitates a tailored approach to treatment, often involving more complicated surgical procedures to repair the urinary tract [12]. The integration of multidisciplinary care, including urology and radiology, is necessary for optimizing outcomes in these cases [13]. This case report adds to the growing body of literature highlighting the importance of individualized care in managing urological trauma complicated by congenital anomalies.

In this context, the present case illustrates a unique convergence of pathologies: a complete ureteral duplication associated with ureteropelvic junction obstruction and ectopic insertion into the seminal vesicle, which predisposed the upper collecting system to chronic hydrocalycosis and subsequent rupture following trauma. The coexistence of these anomalies explains both the unusual clinical presentation and the mechanism leading to retroperitoneal urinoma formation.

The study was conducted in accordance with the Declaration of Helsinki and was approved by the Ethics Committee of “Sf. Apostol Andrei” County Emergency Hospital, Constanța (approval no. 2771/16 January 2024). Written informed consent was obtained from the patient for publication of this case report and any accompanying images.

## 2. Case Report

A 22-year-old male with no prior significant medical history presented to the Emergency Department following a traumatic fall from a height of approximately 80 cm (the fourth step of a staircase). The patient reported a general deterioration in condition, right lumbar pain, right hypochondrial and flank pain, nausea, and abrasions on the right lumbar region and left epicranial occipital abdomen. These symptoms began immediately following the trauma.

Given the severity of the findings, laboratory tests (Table S1 included in Supplementary Materials) were performed on admission including a complete blood count.

The blood test results from hospital admission reveal that the patient’s organ functions are largely within normal limits, indicating a stable condition following recent trauma and surgical interventions. Renal function appears preserved, with creatinine and urea levels within acceptable ranges, though the slight elevation in creatinine warrants ongoing monitoring. Liver function tests, including AST and ALT, also fall within normal limits, suggesting no significant hepatic injury or inflammation. Hematological parameters, such as Hemoglobin, Hematocrit, RBC, WBC, and platelet counts, are all within normal ranges, indicating normal red blood cell production, adequate oxygen-carrying capacity, and no signs of thrombocytopenia or bone marrow suppression. The normal WBC count, alongside previously elevated CRP, suggests that any acute inflammatory or infectious process is likely under control.

Immune function tests, particularly lymphocyte and neutrophil percentages, are also within normal limits, indicating a balanced immune response without evidence of ongoing bacterial infection. The INR value of 1.21 confirms that the patient’s clotting function is normal, reducing the risk of bleeding or thrombosis.

A more detailed anamnesis revealed that the patient had experienced a long-standing history of altered seminal fluid aspect during ejaculation over several years prior to the traumatic event. This symptom had not been previously investigated. In retrospect, this clinical detail is suggestive of an underlying ectopic ureteral insertion into the seminal vesicle and provides additional support for the congenital anatomical anomaly identified during imaging and surgical exploration.

**Figure 1:**
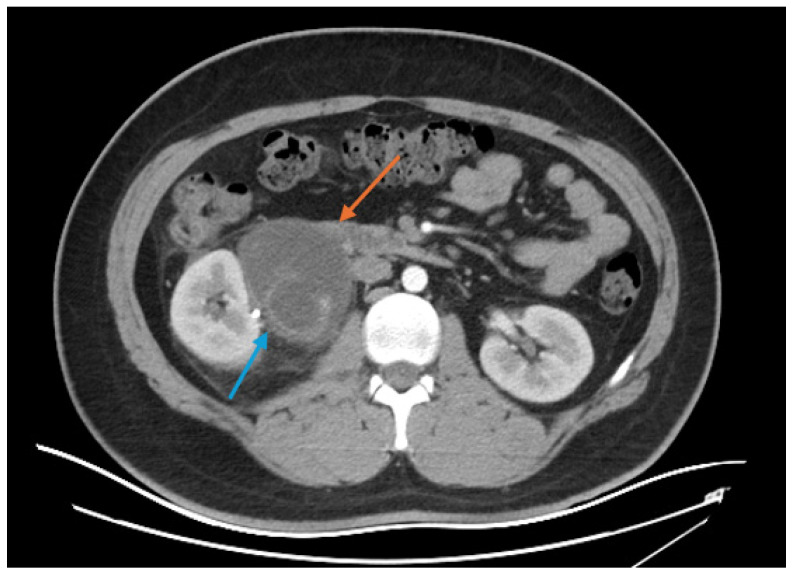
Cross-sectional CT scan of the abdomen before the 1st surgery: abnormal fluid collection around the kidneys (orange arrow); rupture of the renal pelvis (blue arrow).

Upon admission, the patient undertook a comprehensive series of CT scans to assess the full extent of the injuries sustained from the fall. The cranial CT scan revealed pericerebral fluid spaces within normal limits for the patient’s age, with no evidence of intracranial or extracranial masses, post-traumatic changes, or midline shifts. The ventricular system appeared symmetric and appropriately sized, suggesting no acute intracranial pathology. However, a small occipital hematoma was identified on the left side, though it was deemed minor and without significant mass effect. The cervical spine CT scan from C1 to T1 was similarly unremarkable, showing no signs of post-traumatic bone lesions or axis deviations, indicating that the cervical spine was structurally intact. Figure 1 shows the CT scan before the 1st surgery.

The image focuses on the area surrounding the kidneys and retroperitoneal space. The kidneys appear to be enhanced with contrast material, allowing for clear visualization of their internal structures. There is an abnormal fluid collection around the kidneys, particularly on the left side. This fluid is likely in the retroperitoneal space, which is consistent with the presence of urine outside the confines of the renal pelvis and ureters. The image suggests that there has been a rupture of the renal pelvis (specifically the part serving the upper calyx), resulting in urine leaking into the surrounding retroperitoneal area. This extravasation is seen as fluid that is not contained within the kidney but rather extends into the surrounding tissues.

In the abdominal and pelvic CT scan, a significant right renal injury was detected. The right kidney, which was slightly enlarged at approximately 100 mm in the bipolar axis, showed a rupture in the superior calyceal stalk with subsequent contrast extravasation, which was visible one hour after contrast administration. This injury led to the formation of a sizable perirenal urinoma, measuring 66 × 55 × 109 mm, extending inferiorly, and a smaller fluid collection in the anterior right pararenal space, measuring approximately 60 × 21 mm. Notably, the patient was found to have a congenital anomaly of complete pyeloureteral duplication on the right side, with the upper caliceal system draining separately. The right kidney and other abdominal organs, including the liver, gallbladder, spleen, pancreas, and adrenal glands, were unremarkable, with no evidence of post-traumatic changes. The urinary bladder was moderately distended but otherwise normal, and the prostate was without significant findings. No intraperitoneal fluid or lymphadenopathy was observed, and the lumbar spine and pelvis were free of traumatic bone lesions.

These findings indicate a complex urological injury with associated congenital anomalies, necessitating careful surgical planning and management to address the renal rupture and prevent further complications.

### 2.1. 1st Surgical Procedure—Exploratory Ureteroscopy

**Figure 2:**
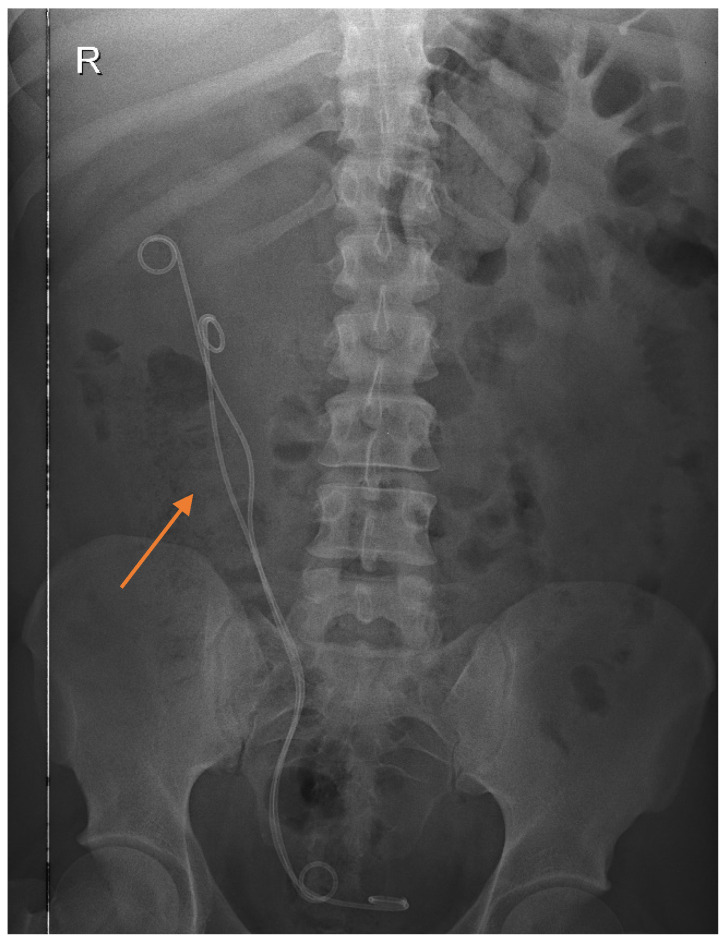
Postoperative radiograph of the abdomen and pelvis following definitive surgical reconstruction (U-trans-U ureteroureterostomy): two double-J ureteral stents, both draining through a single right ureteral orifice into the urinary bladder. The use of dual stenting was required intraoperatively to guide the termino-lateral anastomosis and to ensure patency of both ureteral segments. The anastomosis zone between the two ureters; medially and laterally, in the iliac portion, thus explaining how postoperatively we have two ureters, U trans U (orange arrow).

The patient had a right ureteroscopy with the insertion of a JJ Cook ureteral stent. This procedure was imposed by the presence of a complete pyeloureteral duplication on the right side, as well as a rupture in the superior calyceal stalk leading to urinoma formation. Ureteroscopy is a minimally invasive procedure that allows direct visualization of the ureter and renal pelvis. In this case, the ureter was found to be singular and narrow, with no pathological processes observed up to the level of the pyelocalyceal junction. However, during the procedure, turbid urine was observed being expelled, indicating the presence of infection or inflammatory debris. Figure 2 shows the U-trans-U ureteroureterostomy following the definitive surgical reconstruction.

The JJ stent, also known as a double-J stent due to its curved ends that prevent migration, was placed to ensure continued drainage of urine from the kidney to the bladder. This stent was used to relieve ureteral obstruction, which can occur due to various causes including trauma, stones, or congenital abnormalities like in this case. The procedure involved inserting the flexible wire of the stent through the urethra into the bladder and then advancing it into the ureter under cystoscopic or fluoroscopic guidance. It was then placed over this wire and positioned so that one end resides in the renal pelvis and the other in the bladder, ensuring that the ureter remains patent and urine can flow freely from the kidney to the bladder, bypassing any obstructions or damaged areas.

During cystoscopic evaluation, only a single right ureteral orifice was identified within the bladder.

### 2.2. 2nd Surgical Procedure

Following the initial surgery, a follow-up CT scan of the abdomen and pelvis with contrast was performed to evaluate the patient’s post-surgical status. Compared to the initial scan from the liver was found to be in its normal anatomical position, with slightly heterogeneous contrast uptake due to the presence of a small hypodense, hypo vascular area (approximately 10 × 5 mm) located subcapsular at the junction of segments IV. This area was most likely consistent with focal steatosis. Importantly, there were no signs of intrahepatic bile duct dilatation, and the gallbladder displayed normal wall thickness and content.

The right kidney, however, continued to reveal significant findings. The kidney was now noted to be slightly larger, measuring approximately 117 mm in its bipolar axis, with an irregular parenchymal thickness and contour. Its sinus was traversed by a band of parenchyma that separated the upper caliceal group, which drained through its own ureter, from the middle and lower caliceal groups, which drained through a separate ureter. Persistent dilation of the upper caliceal system was observed, along with a 17 mm defect in the parietal continuity at the level of the renal pelvis, where contrast extravasation was noted 30 min post-injection. There was also a demarcated fluid collection (57 × 75 × 140 mm) surrounding the renal pelvis and the upper ureter, indicating an ongoing urinoma that had progressed compared to the previous examination. The upper ureter was significantly dilated, with a maximum diameter of approximately 13 mm and a thickened, iodine-enhanced wall, showing gradual narrowing in the proximal third of the pelvic segment without any discernible obstruction. Moreover, there was a suspicious communication between the distal part of this ureter and the right seminal vesicle, raising the concern of a possible ureteral-seminal vesicle fistula. The presence of the previously placed Cook stent was confirmed, extending from the inferior caliceal group to the bladder.

Open surgical exploration confirmed the presence of complete right ureteral duplication. The lower pole ureter drained orthotopically into the bladder, while the upper pole ureter followed an ectopic course toward the right seminal vesicle. This anatomical configuration explained both the absence of a second ureteral orifice during cystoscopy and the imaging suspicion of an abnormal communication with the seminal vesicle.

**Figure 3:**
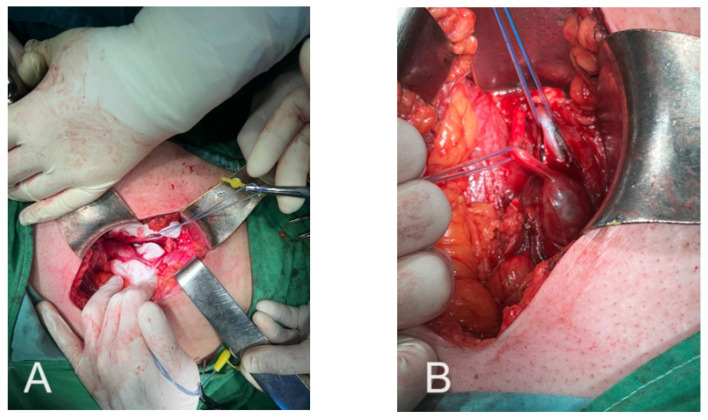
(**A**,**B**): Double malformation: pyeloureteral junction syndrome on the upper foot; pyeloureteral duplicity.

The second surgical procedure was performed to address the ongoing complications related to the patient’s difficult urological condition. The surgery involved a right ilio-inguinal incision to access the retroperitoneal space. Upon entering this area, a significant fluid collection was evacuated, which had formed due to the ongoing urinoma, and inflammatory changes were noted in the surrounding adipose tissue. The surgical exploration revealed the presence of two ureters on the right side, confirming the diagnosis of a complete ureteral duplication (Figure 3).

One of these ureters had been previously stented during the first surgery, while the other ureter was identified with a congenital malformation consistent with a pyeloureteral junction syndrome affecting the upper pole of the kidney. This malformed ureter was carefully dissected and incised distally as much as possible, and then ligated to prevent further complications.

**Figure 4:**
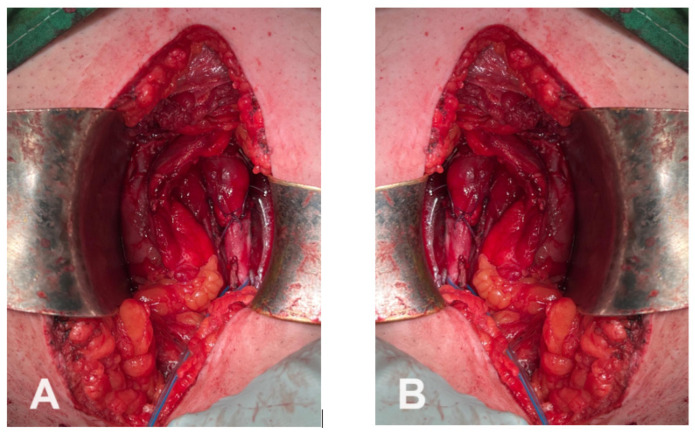
(**A**,**B**): The Anastomosis procedure.

Definitive reconstruction consisted of ureterolysis, dismembered pyeloplasty using the Hynes–Anderson technique for the obstructed upper pole, and termino-lateral ureteroureterostomy (U-trans-U), with the lateral ureter anastomosed into the medial ureter. The procedure was performed over double-J ureteral stents placed in both ureters, which were exteriorized through the single right ureteral orifice to ensure adequate drainage and to guide the anastomosis (Figure 4).

To restore proper urinary drainage, the surgical team excised the malformed pyeloureteral junction and performed a termino-lateral ureteroureterostomy using the U trans U technique. This involved creating a side-to-end anastomosis between the proximal end of the remaining healthy ureter and the distal segment of the stented ureter, ensuring that the upper pole of the kidney was properly drained through the newly created junction. The procedure was facilitated by the use of the previously placed stent as a guide to ensure the correct positioning and patency of the anastomosis. Additionally, a lumbar drain was placed to manage any postoperative fluid accumulation, and a viscerolysis was performed to free up any adhesions that had formed in the retroperitoneal space, likely due to the previous surgery and ongoing inflammation.

Table S2 (Supplementary Material) shows the elevated C-Reactive protein level of 16.15 mg/dL, well above the normal upper limit of 0.5 mg/dL, indicating a significant inflammatory response, likely related to the recent trauma and associated complications such as the urinoma. However, the WBC remains within normal limits, suggesting that while inflammation is present, there is no overt systemic infection at this point. This indicates that the patient’s immune response is active but controlled, with no current signs of overwhelming infection.

The renal function markers, including BUN and creatinine, were within normal ranges, suggesting that the kidneys are functioning adequately despite the trauma and subsequent surgical interventions. Additionally, hematological parameters such as haemoglobin, hematocrit, and platelet counts are stable, indicating that the patient is not experiencing significant blood loss, anaemia, or coagulopathy.

Microscopic analysis of the biopsy revealed significant findings in both tissue fragments collected. The ureteral lining in both samples displayed areas of focal ulceration and reactive changes. Additionally, the surrounding connective tissue contained congested blood vessels, focal haemorrhage, and a mild diffuse infiltration of lymphocytes, indicative of an inflammatory response. These changes suggest that the tissue has been subjected to inflammation and possibly trauma, leading to the observed pathological alterations.

### 2.3. Post-Operatory Evolution

Postoperatively, the patient demonstrated a favourable recovery. Table S3 (Supplementary Material) shows the blood results following the second surgery. This set of blood test results show a mixed picture of improvements and areas of concern. Notably, the CRP level has decreased from 16.15 mg/dL to 10.13 mg/dL, indicating a reduction in the inflammatory response, likely reflecting the effectiveness of the ongoing treatment. However, the BUN and Urea levels have significantly increased, suggesting a potential decline in renal function or ongoing stress on the kidneys. Additionally, the slight decrease in Hemoglobin and Hematocrit levels may reflect ongoing fluid shifts or mild anaemia, which warrants further monitoring.

**Figure 5:**
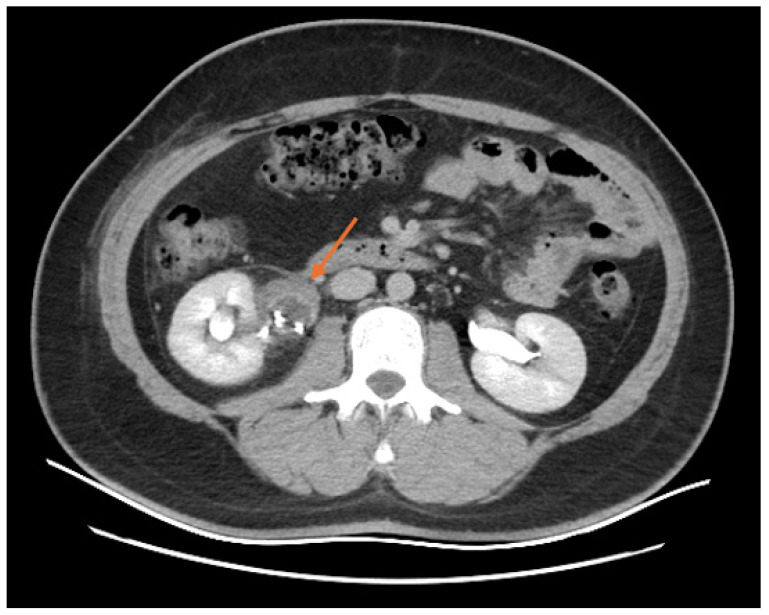
Thirty-days follow-up CT scan of the abdomen: ureteral stent (orange arrow).

The patient received antibiotics, analgesics, and hydro-electrolytic rebalancing to support healing and prevent complications. The patient was discharged in an improved general condition, afebrile, with stable vital signs and no significant complications. He reported easy and pain-free urination, indicating successful resolution of the urinary tract issues. A follow-up CT scan was performed after the endoscopic insertion of the ureteral JJ stent (Figure 5), given the complexity of the right urinary tract malformation, in order to reassess urinary drainage and evaluate the evolution of the urinoma.

**Figure 6:**
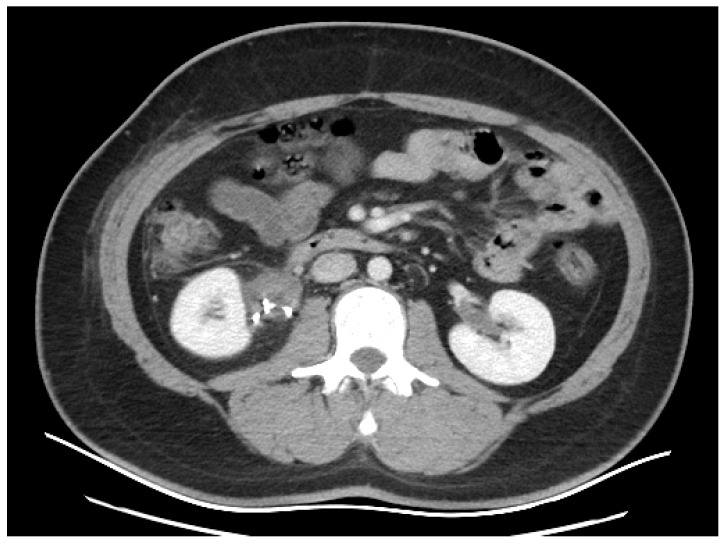
Thirty-days follow-up CT scan of the abdomen.

The image shows the presence of the ureteral stent, with the upper coil of the stent visible within the kidney, indicating that it is correctly placed. The image suggests that there has been a resolution of the urinoma. There are no significant signs of fluid accumulation around the kidneys, indicating that the postoperative condition, possibly due to a previously treated urine leak, has improved. The image provided in Figure 6 is taken during the urographic phase, which occurred about 5 min after the administration of contrast material to evaluate the urinary system.

The kidneys are clearly visualized, with contrast material filling the collecting systems. This enhancement allows for detailed imaging of the kidneys, ureters, and bladder to assess the function and structure of the urinary system.

The second procedure was well-tolerated as the patient experienced only a mild burning sensation during urination, blood-tinged urine, but these symptoms disappeared within a few days. The stent was intended to be a temporary solution and it was removed after 21 days. In this case, the stent placement was fundamental for managing the urological injury and ensuring that the patient’s renal function was maintained while further surgical planning could be undertaken.

The patient was given specific follow-up instructions, including returning to the hospital’s Pathology Service after 45 days to obtain the histopathological results and to have a urological consultation based on those findings. Additionally, a follow-up appointment was scheduled in 30 days for the removal of the JJ stent, which was placed during the 1st surgical intervention, and to undergo a repeat imaging study, specifically a Uro-CT, to ensure the stability and proper functioning of the urinary system.

## 3. Discussion

Renal trauma is commonly stratified using the American Association for the Surgery of Trauma (AAST) renal injury grading system, which classifies injuries from Grade I to Grade V based on imaging findings and the extent of parenchymal and vascular damage [14]. Injuries involving disruption of the collecting system with urinary extravasation are typically categorized as Grade IV renal trauma [15].

In the present case, the imaging findings initially suggested a similar pattern due to the presence of urinary extravasation and retroperitoneal urinoma formation. However, detailed CT evaluation, including delayed excretory phase imaging at 10 and 60 min, revealed that the mechanism differed from classical traumatic renal injury. The patient had a previously undiagnosed congenital anomaly consisting of complete ureteral duplication associated with ureteropelvic junction obstruction (UPJO) and ectopic insertion of the duplicated ureter into the seminal vesicle. This anatomical configuration resulted in chronic dilation of the upper collecting system, with hydrocalycosis of the upper pole.

The urinoma therefore resulted from rupture of the upper pole pyelon secondary to chronically increased intrapelvic pressure, rather than from a traumatic parenchymal laceration. Importantly, no renal vascular injury, segmental infarction, or active bleeding was identified. Consequently, although the presence of urinary extravasation resembles features of AAST Grade IV injury, the underlying pathophysiology in this case represents rupture of a pathologically dilated collecting system within a congenital anomaly rather than classical renal parenchymal trauma. This distinction has important implications for management, as treatment was directed primarily toward urinary diversion and correction of the underlying anatomical abnormality rather than treatment of a traumatic renal laceration.

The presence of a single ureteral orifice on cystoscopy, in contrast with complete ureteral duplication identified intraoperatively, is explained by the ectopic insertion of the upper pole ureter outside the bladder, in this case toward the seminal vesicle.

The management of traumatic urinomas, particularly when complicated by congenital anomalies like pyeloureteral duplication, necessitates a nuanced and individualized approach [16]. Similar cases in the literature emphasize the critical importance of early detection and intervention. For instance, a case report of a urinoma following a kidney biopsy preseted by Limwattana et al. emphasized that delayed treatment can lead to severe complications such as abscess formation, hydronephrosis, and obstructive uropathy, which can result in chronic kidney failure if not promptly managed [17].

Current management strategies for collecting system injuries are guided by recommendations from the European Association of Urology (EAU) [18] and the American Urological Association (AUA) [19]. In hemodynamically stable patients with urinary extravasation, these guidelines generally recommend initial conservative management with urinary diversion, most commonly achieved through ureteral stenting or percutaneous drainage [18,19].

In the present case, the initial intervention followed these principles. Cystoscopy identified a single right ureteral orifice, which was subsequently explored ureteroscopically to confirm ureteral integrity. A double-J ureteral stent was then inserted, providing urinary diversion and decompression of the collecting system, thereby facilitating drainage of the lower two-thirds of the right kidney.

However, further imaging revealed a previously undiagnosed congenital anomaly consisting of complete ureteral duplication associated with UPJO and ectopic insertion of the duplicated ureter into the seminal vesicle. Because of this complex anatomical configuration, urinary diversion alone could not definitively address the underlying pathology. After stabilization, definitive surgical treatment was therefore performed, consisting of meticulous ureterolysis, resection of the ureteropelvic junction, and termino-lateral ureteroureterostomy between the duplicated ureters over a JJ stent.

This staged strategy, initial decompression followed by reconstructive correction of the anatomical abnormality, remains consistent with the principles of guideline-based management, although the rare congenital malformation required individualized surgical adaptation beyond standard algorithms.

Several cases of ectopic ureteral insertion into the seminal vesicle have been reported in the literature, although the condition remains extremely rare [20,21,22,23,24]. In many cases, this anomaly is associated with other developmental abnormalities of the mesonephric duct derivatives, such as renal dysplasia, ureteral duplication, or variants of Zinner syndrome.

For example, Gršković et al. described a patient with a duplicated collecting system in which the ectopic ureter drained into the seminal vesicle and presented as a large retroperitoneal cyst associated with infertility, ultimately requiring surgical excision [25]. Similarly, Zaliznyak et al. reported a rare variant of Zinner syndrome characterized by an ectopic ureter inserting into a cystic seminal vesicle, presenting with recurrent epididymitis and treated by robotic nephroureterectomy [26].

In most reported cases, patients present with chronic genitourinary symptoms such as recurrent infections, hematospermia, infertility, or pelvic pain, while many anomalies remain undiagnosed until adulthood. Compared with these reports, the present case is notable because the congenital anomaly remained previously undetected and manifested acutely following trauma, resulting in rupture of a chronically dilated collecting system and retroperitoneal urinoma formation. To our knowledge, trauma-triggered rupture of a duplicated collecting system with ectopic ureteral insertion into the seminal vesicle has been rarely documented, emphasizing the diagnostic and therapeutic challenges associated with such complex.

When congenital anomalies like pyeloureteral duplication are present, the surgical approach must be meticulously planned to accommodate the unique anatomical challenges [27]. Literature on surgical correction of ureteral duplications highlights the necessity of precise anastomosis and stenting to maintain proper urinary drainage and prevent recurrent obstructions or infections [28]. In the present case, the termino-lateral uretero-ureteral anastomosis and subsequent stenting were vital in restoring normal urinary function and preventing further complications.

A particular feature of this case was the suspected fistulous communication between the lateral ureter and the right seminal vesicle on CT imaging, together with the absence of a second right ureteral orifice during cystoscopy. This suggests an ectopic course of the ureter draining the upper pole moiety in the setting of complete ureteral duplication. While similar mechanisms are described in the EAU and AUA guidelines, they usually involve ectopic ureteral insertion within the bladder, whereas extravesical drainage toward the seminal vesicle is much rarer. Definitive surgical management therefore consisted of ureterolysis, pyeloplasty with resection of the ureteropelvic junction, and termino-lateral ureteroureterostomy over a JJ stent to restore urinary drainage. Complicated cases involving multiple congenital anomalies often require a combination of surgical techniques. Reports have demonstrated that procedures like ureteral reimplantation can be effectively employed to address these urological conditions [29]. The use of imaging techniques such as Uro-CT for postoperative follow-up is consistently recommended in the literature to monitor for residual or recurrent issues, ensuring the success of the surgical intervention and the patient’s long-term recovery [30].

Congenital anomalies of the urinary tract may predispose the genitourinary system to injury even after relatively minor trauma [31]. In the present case, the patient had a congenital malformation consisting of complete pyeloureteral duplication, associated with ureteropelvic junction obstruction of the upper pole moiety and a fistulous communication between the lateral ureter and the right seminal vesicle.

Although ureteral duplication is a relatively well-described condition, the coexistence of upper pole ureteropelvic junction obstruction and extravesical ectopic drainage toward the seminal vesicle represents a rare anatomical variant [32].

According to the EAU and AUA guidelines [18,19] initial management of collecting system injuries with urinary extravasation generally involves urinary diversion using a double-J ureteral stent, with or without percutaneous drainage when a significant urinoma is present. In many cases, this approach allows stabilization of the patient and resolution of the leak.

In the present case, ureteral drainage with a JJ stent was performed as the initial therapeutic step. However, definitive treatment required correction of the underlying congenital anomaly. The patient had complete ureteral duplication with ureteropelvic junction obstruction of the upper pole moiety, while the second ureter followed an ectopic course toward the seminal vesicle, which prevented endoscopic transurethral access.

Therefore, definitive surgical management consisted of extended ureterolysis, resection of the ureteropelvic junction (pyeloplasty) of the upper pole pelvis, and termino-lateral ureteroureterostomy between the duplicated ureters over a JJ stent, restoring adequate urinary drainage.

This case aligns with findings from other case reports that emphasize the importance of a multidisciplinary approach in managing traumatic and congenital urological conditions [33]. Early surgical intervention, careful planning, and diligent postoperative care are key factors that contribute to favorable outcomes in these cases [34]. Continued documentation and study of such cases are necessary to refine surgical techniques and enhance patient care in similar clinical scenarios [35].

## 4. Limitations

This case report focuses on a single patient with a rare combination of traumatic injury and congenital pyeloureteral duplication, limiting the generalizability of the findings to broader populations. The unique nature of the case means that the surgical outcomes and recovery process may not be applicable to other patients with similar conditions.

The follow-up period in this study was relatively short, focusing primarily on the immediate postoperative outcomes. Long-term follow-up is necessary to assess the durability of the surgical repair, the resolution of the urinoma, and the patient’s overall renal function over time. Another limitation is that this study does not include a comparison with other management strategies, such as conservative treatment or alternative surgical techniques, which could provide insights into the relative effectiveness of the chosen surgical approach.

Moreover, the study did not include a detailed functional assessment of the affected kidney post-surgery. Evaluating renal function through imaging and laboratory tests over a longer period could provide a more comprehensive understanding of the surgery’s impact on the patient’s overall kidney health.

## 5. Conclusions

This case report highlights the successful management of an intricate urological condition involving traumatic urinoma and congenital pyeloureteral duplication. The patient underwent two surgical interventions, including ureteral stenting and uretero-ureteral anastomosis, which led to a positive outcome with stable renal function and resolution of the urinoma. The case points out the importance of early diagnosis, tailored surgical planning, and diligent postoperative care in managing such cases. Despite the limitations, this case contributes valuable insights into the management of similar urological conditions, emphasizing the need for individualized treatment approaches and long-term follow-up to ensure optimal patient outcomes.

## Data Availability

The original contributions presented in this study are included in the article. Further inquiries can be directed to the corresponding author.

## Supplementary Materials

The following supporting information can be downloaded at: https://www.mdpi.com/article/10.3390/diagnostics16081132/s1, Table S1: Laboratory tests on hospital admission; Table S2: 2nd post-op blood results; Table S3: 3rd post-op blood results.
