# Supplementary material for: Dual Challenges: Addressing Post-Traumatic Retroperitoneal Urinoma in the Context of Pyeloureteral Duplication
Source: Diagnostics (Basel). 2026 Apr 9;16(8):1132. doi: 10.3390/diagnostics16081132 (PMC13114467; doi:10.3390/diagnostics16081132)
Supplement: Supplementary file 1 [file diagnostics-16-01132-s001.zip › diagnostics-4146475-supplementary.pdf]

SUPPLEMENTARY MATERIAL

Table S1. Laboratory tests on hospital admission

Test	Result	Normal range	Unit
Creatinine	1.055368	0.5 - 1.2	mg/dL
Basophils	0.04	0.01 - 0.08	thous/µL
Basophils (%)	0.4	0.1 - 1.2	%
Eosinophils	0.28	0.04 - 0.50	thous/µL
Eosinophils (%)	2.5	0.7 - 6	%
Hematocrit	40.5	36 - 54	%
Hemoglobin	14.7	12 - 18	g/dL
Lymphocytes	33.8	20 - 50	%
Mean corpuscular hemoglobin	27.8	27 - 34	pg
Monocytes	0.68	0.24 - 0.82	thous/µL
Monocytes (%)	6.2	4.7 - 12.5	%
Mean platelet volume	10.2	7.4 - 11	fL
Neutrophils	6.32	1.5 - 7	thous/µL
Neutrophils (%)	57.1	34 - 71	%
Platelet distribution width	10.9	9 - 17	fL
Platelet large cell ratio	25.0	13 - 43	%
Platelet count	189	150 - 450	thous/µL
Red blood cell count	5.28	3.5 - 6	mill/µL
Red cell distribution width-coefficient of variation	11.9	11.5 - 16	%
Aspartate aminotransferase	34.2282	2 - 40	U/L
Alanine aminotransferase	37.2497	2 - 40	U/L
Urea	33.39304	0 - 50	mg/dL
Prothrombin time	1.21	-	Ratio

Table S2. 2st post-op blood results

Test	Result	Normal range	Unit
Blood urea nitrogen	17	≤20	mg/dL
Creatinine	0.76	≤1.2	mg/dL
C-Reactive protein	16.15	≤0.5	mg/dL
Urea	37	10-50	mg/dL
Hematocrit	39.2	39-49	%
Hemoglobin	13.8	13.2-17.3	g/dL
Red blood cell count	4.95	4.3-5.7	mill/μL
White blood cell count	4.92	4-10	thous/μL
Platelet count	206	150-450	thous/μL

Table S3. 3nd post-op blood results

Test name	Result	Normal range	Unit
Blood Urea Nitrogen	41	≤20	mg/dL
Alanine Aminotransferase	11	<41	U/L
Aspartate Aminotransferase	15	<40	U/L
Creatinine	0.62	≤1.2	mg/dL
C-Reactive Protein	10.13	≤0.5	mg/dL
Glucose	89	70-100	mg/dL
Potassium	4.6	3.5-5.1	mmol/L
Sodium	139	136-145	mmol/L
Bicarbonate	22.2	22-26	mEq/L
Urea	81	10-50	mg/dL
Basophils	0.06	0.01-0.08	thous/μL
Basophils (%)	0.6	0.1-1.2	%
Eosinophils	0.11	0.04-0.50	thous/μL
Eosinophils (%)	1.1	0.7-6	%
Hematocrit	38.6	36-54	%
Hemoglobin	13.5	12-18	g/dL
Mean corpuscular hemoglobin	27.7	27-34	pg/cell
International normalized ratio	1.22	-	Ratio
Red blood cell count	4.95	4.3-5.7	mill/μL
White blood cell count	6.7	4-10	thous/μL
Platelet count	157	150-450	thous/μL
Mean platelet volume	10.7	7.4-13	fL
